# Ocular Symptoms in Pre- and Perimenopausal Woman Infected with *Demodex* spp.

**DOI:** 10.3390/pathogens14030297

**Published:** 2025-03-20

**Authors:** Danuta Kosik-Bogacka, Natalia Łanocha-Arendarczyk, Renata Pilarczyk, Daria Schneider-Matyka, Karolina Kot, Konrad Grzeszczak, Joanna Pyzia, Elżbieta Grochans

**Affiliations:** 1 Department of Biology, Parasitology and Pharmaceutical Botany, Pomeranian Medical University in Szczecin, Powstanców Wielkopolskich 72, 70-111 Szczecin, Poland; natalia.lanocha.arendarczyk@pum.edu.pl (N.Ł.-A.); karolina.kot@pum.edu.pl (K.K.); 2 Laboratory of Biostatistics, Bioinformatics and Animal Research Faculty of Biotechnology and Animal Breeding, West Pomeranian University of Technology in Szczecin, 71-270 Szczecin, Poland; renata.pilarczyk@zut.edu.pl; 3 Department of Nursing, Pomeranian Medical University in Szczecin, Żołnierska 48, 71-210 Szczecin, Poland; daria.schneider.matyka@pum.edu.pl (D.S.-M.); elzbieta.grochans@pum.edu.pl (E.G.); 4 Department of Medical Analytics, Pomeranian Medical University in Szczecin, Powstanców Wielkopolskich 72, 70-111 Szczecin, Poland; k.grzeszczak@kidl.org.pl; 5 Department of Ophthalmology, Independent Public Health Care Complex in Gryfice, Niechorska 27, 72-300 Gryfice, Poland; joanna11224@wp.pl

**Keywords:** *Demodex folliculorum*, *Demodex brevis*, pre- and perimenopausal women

## Abstract

The aim of this study was to determine the subjective ocular symptoms in pre- and perimenopausal women infected with *Demodex folliculorum* and *D. brevis*. Eyelashes were taken from pre- and perimenopausal women aged from 45 to 69 years (n = 253) and younger women aged from 3 to 40 (n = 204) from the West Pomeranian Voivodeship located in Poland. The prevalence of mites was analyzed according to age and subjective ocular symptoms. *Demodex* spp. were detected in 75/253 (29.64%) of pre- and perimenopausal women and in 25/204 (12.25%) of younger women. *Demodex folliculorum* or *D. brevis* was observed in 72/252 (28.45%) and 1/253 (0.4%) of pre- and perimenopausal women, respectively, but the coinfection of *D. folliculorum* and *D. brevis* was noted in 2/253 (0.79%) of women. In young women, only *D. folliculorum* was detected. Single *Demodex* spp., multiple parasites, and numerous mites were reported in about 75%, 17%, and 8% of the examined women, respectively. There was a statistically significant relationship between *Demodex* spp. infestation and the occurrence of dryness of the eyes. Changes occurring in the female body during the pre- and perimenopausal periods lead to an increased incidence of *Demodex* spp. infestation. Women who report dryness of the eyes should have their eyelashes microscopically examined for *Demodex* spp.

## 1. Introduction

*Demodex* spp. are cosmopolitan mites occurring in many species of mammals. Human demodicosis is a parasitic disease caused by two species of mites, *Demodex folliculorum* (Simon, 1842) and *D. brevis* (Akbulatova, 1963) (Acariformes: Prostigmata, Demodicidae) [1]. *Demodex folliculorum* is the most often noted ectoparasite of hair follicles and Zeiss glands of eyelid margin, and has significant implications for ocular health [2]. *Demodex brevis* resides solitarily in the meibomian and sebaceous glands around the eyelash follicles [3]. *Demodex* infection can be transmitted directly by close contact with infected people. In addition, it can be transmitted indirectly by contaminated towels, combs, blankets, bath sponge, and night clothes [4]. *Demodex* spp. infestation usually remains asymptomatic and may have a pathogenic role only when present in high densities or because of immune imbalance [5]. Demodecosis has been linked to various disorders such as rosacea and pityriasis, but also metabolic syndrome, and diabetes without a proven mechanism [1,6]. *Demodex* mites have been implicated in anterior and posterior blepharitis, blepharoconjunctivitis, and blepharokeratitis [3]. However, the etiologic contribution of *Demodex* spp. to ocular diseases remains unconfirmed [7]. The severity of the pathology varies depending on the age factor and the state of the immune system [2]. Moreover, skin phototype, sunlight exposure, alcohol intake, smoking, and stress may influence the development of ocular demodicosis [1]. Demodicosis is highly age-dependent; it is observed in ~80% of the population aged 60 years and 100% of the general population aged above 70 years [8].

Structural and functional changes occur during the postmenopausal period, including ocular changes, which are associated with estrogen (E2) deficiency [9,10]. Additionally, there are hormonal imbalances during this time and tear production deteriorates, and ocular tissues become vulnerable to pathogens [11]. E2 has a proinflammatory effect on meibomian glands, which results in reduced lipid production from the meibomian glands located on the eyelids. Some authors have suggested that the meibomian gland orifices are occluded, which increases the eyelids’ exposure to microorganisms (perhaps also to *Demodex* spp.) due to the impaired circulation there. It is possible that this is the path by which the induction of blepharitis, characterized by inflammation and infection in the eyelids, may develop [10].

*Demodex* spp. are an important ophthalmological problem, especially in an aging population [12]. Therefore, the aim of this study was to determine the subjective ocular symptoms in pre- and perimenopausal women infected with *D. folliculorum* and *D. brevis*.

## 2. Materials and Methods

The research was carried out from June 2017 to December 2019. It was approved by the Bioethics Committee of the Pomeranian Medical University in Szczecin (KB-0012/181/13, KB-0012/100/17, and KB-0012/160/19), and conducted in accordance with the World Medical Association’s (WMA) Declaration of Helsinki on the ethical principles of medical research involving human subjects. The study included 253 women aged from 45 to 60 years (mean age: 53.08  ±  5.04 years) from the West Pomeranian Voivodeship in Poland, who had volunteered after receiving information about the study from local papers and information posters in public places. The study sample was described in detail by Szkup et al. [13]. The criteria for inclusion in pre- and perimenopausal women were as follows: female sex, age between 45 and 60 years, the lack of current cancerous, psychiatric, or inflammatory diseases, and deliberate written consent to take part in the study. In addition, we examined 204 young women aged from 19 to 40 (mean age: 30.60 ± 6.72 years). The criteria for inclusion in the young women were as follows: female sex and age between 18 and 60 years.

Each participant had to fill out a questionnaire to provide sociodemographic data (age) and information about subjective ocular symptoms. Four eyelashes, each from the right and left upper eyelids, were taken from the female subjects in both groups using sterile tweezers. The eyelashes were then placed between two basal slides and labeled with a code number. The material was evaluated using an OLYMPUS CX21LED light microscope (Olympus, Tokyo, Japan) at a ×40 magnification. Infestation was defined as the presence of eggs, larvae, or mature forms of *Demodex* spp. on the eyelashes; species identification was based on Desch and Nutting [14].

The intensity of *Demodex* spp. infestation was assessed at a magnification of ×40 and categorized using three levels of parasite load: low—single (≤2) mites in almost every field of vision; medium—3–9 mites in every area of vision; and high—≥10 mites in every field of vision.

The results were analyzed using Statistica 13.0 (TIBCO Software Inc., Santa Clara, CA, USA). A non-parametric chi-square test or Fisher’s Exact Test was applied, and the level of significance was set at *p* < 0.05. Correspondence analysis (CA) was also performed to visualize the relationship between *Demodex* spp. infection and the occurrence of ocular symptoms in pre- and perimenopausal women.

## 3. Results

*Demodex* spp. were detected in 29.64% of pre- and perimenopausal women and in 12.25% of young women (Table 1). In pre- and perimenopausal women, *D. folliculorum* was observed in 28.45% of women, while *D. brevis* was noted only in 0.4% of female patients. Two participants (0.79%) had coinfection of *D. folliculorum* and *D. brevis*. In young women, only *D. folliculorum* was detected. Pre- and perimenopausal women demonstrated a significantly higher prevalence of *Demodex* spp. (χ^2^ = 20.0; *p* < 0.001).

**Figure 1:**
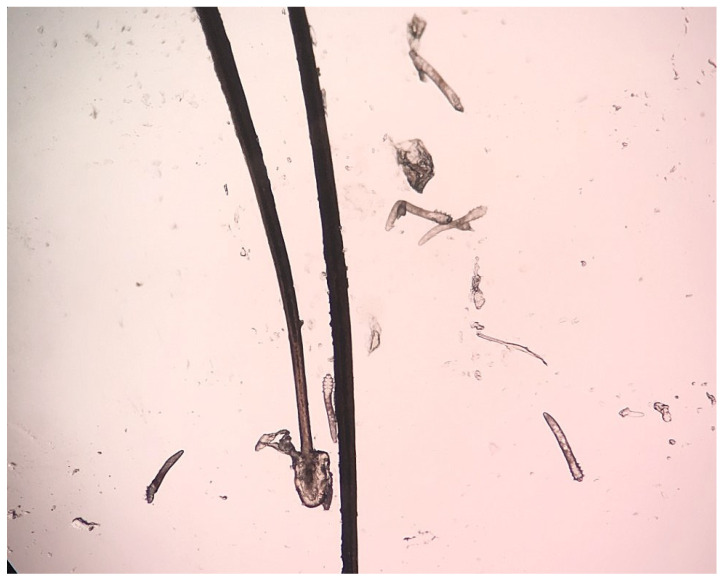
Multiple developmental forms of *Demodex folliculorum*.

The mature *D. folliculorum* forms were most often detected, while larvae and nymphs were found less frequently. In woman infected with *D. brevis*, only the mature forms were found. Eggs were noted in three pre- and perimenopausal women infected with *D. folliculorum*. Cylindrical dandruff was found in all infected women (Figure 1).

Single *Demodex* spp. (up to two developmental forms), multiple parasites (from three to nine developmental forms) (Figure 1), and numerous mites (>ten developmental forms) were reported in about 75%, 17%, and 8% of the examined women from both groups, respectively (Table 2). No significant differences were noted in the intensity of *Demodex* spp. infestation in analyzed groups.

Data concerning the prevalence of ophthalmological symptoms, including itching, tearing, eyelid redness, dryness of the eyes, foreign body sensation, and excessive eyelash loss, were collected from pre- and perimenopausal women (Table 3). Due to differences in the number of women in the groups according to the intensity of *Demodex* spp. infestation, this variable was not taken into account in the statistical analysis. Itching of the eyes was observed in 62.67% and 57.3% of infected and non-infected with *Demodex* spp. women, respectively. There was no statistically confirmed relationship between *Demodex* spp. infection and the occurrence of itching of the eyes (χ^2^ = 0.63; *p* = 0.43). Tearing was noticed in 48.0% of pre- and perimenopausal women infected with *Demodex* spp. This symptom occurred mainly in women who had two or less developmental forms of *Demodex* spp. Tearing was reported also in 34.27% of non-infected women. A statistically significant relationship was noted between *Demodex* spp. infection and tearing (χ^2^ = 4.21; *p* = 0.04). Redness of the eyelids was found in 30.67% of women infected with *Demodex* spp. This symptom occurred mainly in women who had two or less developmental forms of *Demodex* spp. Redness of the eyelids was also found in 16.85% of the non-infected with *Demodex* spp. pre- and perimenopausal women. A statistically significant relationship was noted between *Demodex* spp. infection and eye redness (χ^2^ = 6.08; *p* = 0.01). Dry eyes were present in 36.0% of pre- and perimenopausal women infected with *Demodex* spp.; usually, this symptom occurred mainly in women who had single parasites and multiple mites (about 40%). Moreover, dry eyes were noted in 22.47% of uninfected patients. A statistically significant relationship was noted between *Demodex* spp. infestation and the occurrence of dryness of the eyes (χ^2^ = 4.96; *p* = 0.03). A foreign body sensation under the eyelids was observed in 18.67% of women infected with *Demodex* spp. and in 14.61% of non-infected participants. There was no statistically confirmed relationship between *Demodex* spp. infection and the occurrence of foreign body sensation (χ^2^ = 0.65; *p* = 0.42).

**Figure 2:**
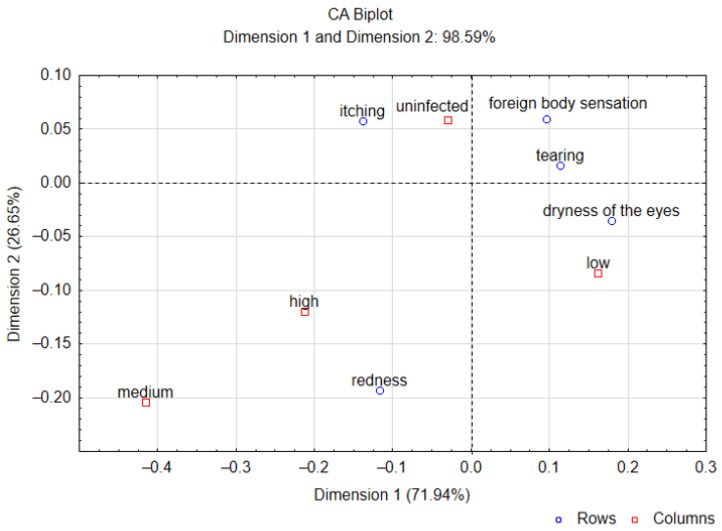
Correspondence analysis (CA) biplot, using ophthalmological symptoms as rows and *Demodex* spp. infection/intensity infection as columns.

Figure 2 presents a correspondence analysis (CA) that visually shows the relationship between *Demodex* spp. infection and the occurrence of five ocular symptoms (itching, tearing, redness of the eyelids, dry eyes, and foreign body sensation) in pre- and perimenopausal women. Due to the low quality of the score for the symptom of excessive eyelash loss (0.48), indicating poor representation in two-dimensional space, a correspondence analysis was performed without taking into account the frequency of this symptom. The CA plot explained a total variance of 98.59%, which accounted for the combination of 71.94% of the variance with Dimension 1 and 26.65% with Dimension 2. Low intensity of *Demodex* spp. infestation was closely associated with the occurrence of dry eyes, while high and medium intensities of *Demodex* spp. infestation were associated with the occurrence of eyelid redness.

## 4. Discussion

*Demodex folliculorum* and *D. brevis* are the most common ectoparasites in humans, especially often found in the elderly [15]. Age is a major risk factor of *Demodex* spp. infection, because changes in the sebum composition and activity of sebaceous glands may facilitate the growth of *Demodex* spp. in the elderly [16]. The increase in *Demodex* spp. infestation with age is probably related to poor eye hygiene [15] and weaker immune system in the elderly [17].

*Demodex* spp. prevalence in women ranges from 30% to 80% [18]. In our investigation, *Demodex* spp. were detected in about 30% of pre- and perimenopausal women and in ~12% of young women. Kemal et al. [19] found *Demodex* spp. in 14.3% to 24.1% of young patients (10–40 years of age) and in 17.4 to 46.2% of old (41–60 years of age) healthy women from Turkey.

Sędzikowska et al. [12] observed that the mean number of *Demodex* spp. in females under 25 years of age and between 26 and 40 years of age is 1.8 and 4.2, respectively, while in females between 41 and 55 years of age, the mean number of parasites is 6.1. In this study, single parasites were the most frequently (75%) found in both studied groups.

*Demodex folliculorum* causes anterior blepharitis, and *D. brevis* causes posterior blepharitis [20,21]. Wesołowska et al. [22] noted that *D. folliculorum* was approximately 2.4 times more common than *D. brevis.* Bohdanowicz and Raszeja-Kotelba [23] noticed that the ratio of *D. folliculorum* to *D. brevis* incidence was 1:10 in women and 1:4 in men. In this study, in pre- and perimenopausal women we observed the separate determination of the presence of *D. folliculorum* alone (29%), *D. brevis* alone (about 0.4%), or the coinfection of *D. folliculorum* and *D. brevis* (0.8%). Moreover, in young women we detected only *D. folliculorum* (12%). Zeytun and Karakurt [17], in patients with and without chronic or ocular disease, detected *D. folliculorum* alone in about 70% of studied participants, both *D. folliculorum* and *D. brevis* in 27% of examined subjects, and *D. brevis* in about 1% of patients. This can be explained by the fact that *D. brevis* resides deep in sebaceous glands, including the meibomian gland, but *D. folliculorum* in the superficial structures of the follicles and glands [24]. Therefore, *D. folliculorum* can be more easily isolated than *D. brevis*. The incidence of *Demodex brevis* confirmed in the study is not real. Therefore, in the future it is advisable to examine not only the eyelashes, but also to analyze the morphology of Meibom’s glands (MGs) along with the clinical picture emerging from physical and subjective examination.

The symptoms of ocular demodicosis are non-specific, and patients are often asymptomatic. *Demodex* spp. infection may be associated with ocular discomfort, including dry eyes, itching and burning of the eyes, foreign body sensation, redness of eyelids, photophobia, blurred vision, increased secretions, and repeated eyelash loss [21,25]. In our study, pre- and perimenopausal women infected with *Demodex* spp. reported itching (~60%), tearing (~40%), redness of the eyelids (~20%), dry eyes (~40%), foreign body sensation (~20%), and eyelash loss (~5%). But Cheng et al. [7] reported that patients with ocular demodicosis suffered from dryness (~70%), itching and irritation (~60%), and pain (~40%). In *Demodex* spp.-infected women from north-western Poland, a statistically significant relationship was noted between *Demodex* spp. infestation and occurrence of dry eye. But Biernat et al. [26] observed that itching and redness were more common in patients infected with *Demodex* spp. with and without blepharitis.

Lee et al. [15] observed positive correlations between *Demodex* spp. symptoms and *Demodex* spp. number in subjects with and without eye diseases. Similarly, Sędzikowska et al. [12] found a positive correlation between *Demodex* number and the frequency of ocular symptoms (itching, redness, watery, and pain).

The present study has some limitations. We did not determine the prevalence of *Demodex* spp. in pre- and perimenopausal women in relation to meibomian dysfunction, conjunctival inflammation, and corneal lesions. Patients infected with *Demodex* spp. should also undergo an ophthalmological examination. The number of volunteers was relatively small, resulting in an inability to conduct statistical analysis.

Based on the data, we concluded that changes occurring in the female body during the pre- and perimenopausal periods lead to an increased incidence of *Demodex* spp. infestation. Women who report dryness of the eyes should have their eyelashes microscopically examined for *Demodex* spp.

## Figures and Tables

**Table 1:** Prevalence of *Demodex* spp. in pre- and perimenopausal as well as young women.

Group	Number/% of Infected Women			
	*Demodex* spp.	*D. folliculorum*	*D. brevis*	Coinfection of *D. folliculorum* and *D. brevis*
pre- and perimenopausal women (n = 253)	75/29.64	72/28.45	1/0.40	2/0.79
young women (n = 204)	25/12.25	25/12.25	0	0
χ^2^ test value	χ^2^ = 20.0; *p* < 0.001	χ^2^ = 20.8; *p* < 0.001		

**Table 2:** The intensity of *Demodex* spp. infestation in analyzed groups (low—≤2 mites in almost every field of vision; medium—3–9 mites in every area of vision; high—≥10 mites in every field of vision).

Group	The Intensity of *Demodex* spp. Infestation		
	Low	Medium	High
pre- and perimenopausal women	56/74.67%	13/17.33%	6/8.0%
young women	19/76.0%	4/16.0%	2/8.0%
χ^2^ test or Fisher’s Exact Test *p* value	*p* = 0.87	*p* = 0.88	*p* = 1.00

**Table 3:** Frequency of subject ocular symptoms in pre- and perimenopausal women.

Symptoms	Uninfected	Infected				χ^2^ Test Value
		Total	The Intensity of *Demodex* spp. Infestation			
			Low	Medium	High	
itching, n (%)	102 (57.30)	47 (62.67)	33 (70.21)	10 (21.28)	4 (8.51)	χ^2^ = 0.63; *p* = 0.43
tearing, n (%)	61 (34.27)	36 (48.00)	31 (86.11)	3 (8.33)	2 (5.56)	χ^2^ = 4.21; *p* = 0.04
redness, n (%)	30 (16.85)	23 (30.67)	16 (69.57)	5 (21.74)	2 (8.70)	χ^2^ = 6.08; *p* = 0.01
dryness of the eyes, n (%)	40 (22.47)	27 (36.00)	24 (88.89)	2 (7.41)	1 (3.70)	χ^2^ = 4.96; *p* = 0.03
foreign body sensation, n (%)	26 (14.61)	14 (18.67)	12 (85.71)	1 (7.14)	1 (7.14)	χ^2^ = 0.65; *p* = 0.42
eyelash loss, n (%)	9 (5.06)	6 (8.00)	4 (66.67)	1 (16.67)	1 (16.67)	χ^2^ = 0.82; *p* = 0.37

## Data Availability

The data presented in this study are available on request from the corresponding author.
